# The potential of using bacteriophages targeting *Salmonella* Dublin in cattle herds

**DOI:** 10.3389/fmicb.2025.1698141

**Published:** 2025-10-29

**Authors:** Sarah Grønlund Jespersen, Veronika Theresa Lutz, Louise Ladefoged Poulsen, Lone Brøndsted

**Affiliations:** Department of Veterinary and Animal Sciences, University of Copenhagen, Copenhagen, Denmark

**Keywords:** *Salmonella* Dublin, cattle, phage therapy, bacteriophages, novel antimicrobial solutions

## Abstract

*Salmonella* Dublin causes severe illness in cattle and humans and can persistently infect cattle herds for years despite comprehensive control efforts. Bacteriophages are viruses that specifically kill bacteria. This paper reviews existing research and discusses the clinical challenges, applications, and research gaps that need to be addressed to explore the potential of bacteriophages in controlling *Salmonella* Dublin in cattle herds. Phages targeting *Salmonella* Dublin have not been systematically isolated for phage therapy applications. However, a few phages infecting *Salmonella* Dublin have been identified and characterized, showing promising survival in conditions relevant to feed and cattle. Still, detailed information about receptors, host range, phage resistance, and co-evolution of phages targeting *Salmonella* Dublin is lacking, but is essential for rational cocktail design. The advantages of phage therapy include its high specificity and narrow host range, which leaves the beneficial microbiota of the animal unharmed. The high clonality of *Salmonella* Dublin within a geographic area can inform the design of targeted phage treatments for different regions. Although the intracellular nature of *Salmonella* Dublin presents a challenge, phages have been shown to internalize at varying rates depending on their size and type. In conclusion, bacteriophages show promise against *Salmonella* Dublin, but the development of specific, well-characterized phages and optimized administration approaches is necessary for phage therapy to realize its full potential.

## Introduction

*Salmonella enterica* subspecies *enterica* serotype Dublin (*Salmonella* Dublin) is one of many serotypes of the *Salmonella* genus, belonging to the *Enterobacteriaceae* family. *Salmonella* infections occur worldwide and in various species, including humans, cattle, pigs, birds, and reptiles. Most serotypes of *Salmonella enterica* have a broad host range; however, some serotypes, including *Salmonella* Dublin, exhibit host adaptation to bovine animals, causing more severe disease than non-adapted serotypes (Holschbach and Peek, 2018; Quinn et al., 2011). *Salmonella* Dublin is challenging due to its persistence in cattle herds (Kudirkiene et al., 2020; Ministeriet for Fødevarer LoF, 2022), its zoonotic potential, causing high mortality in humans (Helms et al., 2003; Do Amarante et al., 2025), and the substantial economic losses associated with infection in cattle herds (Nielsen et al., 2013). Despite ongoing eradication programs and biosecurity measures, *Salmonella* Dublin remains a persistent issue (Kudirkiene et al., 2020; Ministeriet for Fødevarer LoF, 2022). Furthermore, some countries are facing problems with multidrug-resistant strains of *Salmonella* Dublin, which complicates antimicrobial treatment (Eyler et al., 2020; Paudyal et al., 2019; Harvey et al., 2017), thus increasing interest in alternative solutions, especially with the emergence of antibiotic-resistant strains (Do Amarante et al., 2025). Bacteriophages, viruses that target and kill their host bacteria as part of their life cycle, offer promising alternatives to antibiotics. Historically, phages have been used therapeutically in humans and animals, but this practice was discontinued in Western countries after antibiotics became widespread. As antimicrobial resistance becomes a greater concern, interest in phage therapy has grown (Loponte et al., 2021).

In this review, we (i) summarize the pathogenesis and epidemiology of *Salmonella* Dublin, (ii) assess current knowledge of phages against this serotype, (iii) examine strategies for phage cocktail design, including modern molecular tools, (iv) evaluate challenges related to stability and administration in cattle, and (v) highlight key gaps and future research directions.

## *Salmonella* Dublin pathogenesis and epidemiology

The clinical manifestations of *Salmonella* Dublin infections in cattle are fever, pneumonia, sepsis, and cause enteric and reproductive diseases (Holschbach and Peek, 2018; Quinn et al., 2011; Nielsen, 2013). Thus, in addition to gastroenteritis, in-utero infections of bovine fetuses often result in abortions (Nielsen, 2013). The most common transmission route of *Salmonella* Dublin in cattle herds is ingesting contaminated food, water, or milk. In exceptional circumstances, the airways and conjunctiva can function as a portal of entry (Nielsen, 2013). Once ingested, *Salmonella* Dublin rapidly adheres to the intestinal mucosal cells and invades into enterocytes in the terminal jejunal and ileal mucosa (Quinn et al., 2011; Nielsen, 2013). Initial adhesion is more likely if the gastrointestinal microbiota is abnormal, compromised, or underdeveloped (Holschbach and Peek, 2018). Genes associated with cattle-specific adaptation are linked to the cell surface and proteins involved in DNA metabolism and catalytic activities (Merkushova et al., 2023), acquired through gene deletions or horizontal gene transfer into their genome (Singh, 2013). While initial cell adhesion is crucial for *Salmonella* Dublin to infect cattle, the infection can become systemic by entering macrophages and draining the local lymph nodes, enabling it to reach the lymph and bloodstream and cause bacteremia. *Salmonella* Dublin can survive and replicate in various tissue types within the host, including macrophages, thereby shielding itself from host defenses (Nielsen, 2013) and antibiotics (Nielsen et al., 2004). It generally favors lymphoid tissues, invading through M-cells, and is mainly found in Peyer’s patches and mesenteric lymph nodes (Holschbach and Peek, 2018). This may contribute to *Salmonella* Dublin’s persistence in cattle herds, as carrier animals harbor a chronic infection in lymph nodes and internal organs, leading to ongoing or periodic bacterial shedding into the environment. Such carriers complicate eradication efforts by serving as hidden sources of infection and may underestimate the prevalence of *Salmonella* Dublin (Nielsen, 2013; Nielsen et al., 2004). Risk factors for becoming a carrier include age, time since calving, season, and herd prevalence, with the highest risk observed in herds experiencing clinical outbreaks and in herds with low prevalence. This is possibly due to lower shedding, thus exposing the animals to lower doses, evading the immune response, and hindering disease elimination (Nielsen et al., 2004).

The core genome of *Salmonella* Dublin strains exhibits a highly region-specific population structure (Fenske et al., 2019). Interestingly, there is a clear geographic separation between different clades (Fenske et al., 2019), and strains from the same locations often display high clonality (Do Amarante et al., 2025; Fenske et al., 2019), as demonstrated in two studies analyzing genomic data of *Salmonella* Dublin in Denmark. The Danish *Salmonella* Dublin population consisted of two major genetically distinct clades and one small cluster (Kudirkiene et al., 2020; Leekitcharoenphon et al., 2023). Closely related strains are often found within the same herd or in herds linked epidemiologically over several years. This indicates that herds that test positive tend to stay infected with the same strain. The clades are geographically specific and unique to the Danish cattle population. Sia et al. (2025) analyzed 1,303 *Salmonella* Dublin genomes and found that distinct evolutionary lineages were strongly associated with geography, antimicrobial resistance profiles, and plasmid types. Similar results were found in other studies in other regions, like in the U. S., thus suggesting regional adaptation and clonality (Fenske et al., 2019).

## Bacteriophages as alternatives to antimicrobials

Bacteriophages (phages) are viruses that depend on bacteria to reproduce and complete their life cycle. In lytic phages, used for various applications, the phage’s life cycle consists of the specific binding to a bacterial host, the replication and expression of phage genes directing the bacterial metabolism to produce phages, and the release of new phages (Loponte et al., 2021; Gencay and Brøndsted, 2019). As bacteria-killing agents, phages can be used as alternatives to antimicrobials. Furthermore, their high specificity makes them ideal antimicrobials against highly clonal pathogens such as *Salmonella* Dublin. Literature describing phages specifically targeting *Salmonella* Dublin is sparsely described (Chandra et al., 2011; Gencay et al., 2019), but phages infecting other *Salmonella* serotypes have been isolated and described more thoroughly in the literature.

## Host range and phage resistance development

Essential for selecting phages for applications is their host range, e.g., the breadth of bacterial strains the phage can infect and thus kill. Many phages have a narrow host range, meaning they are only efficient against a small subset of the diversity of the target bacteria. Statistical analysis of phenotypic and genetic data on a collection of phages infecting *Salmonella* showed that the phage genus and the bacterial receptor are the two major determinants of host range (Gencay et al., 2019). Phage genome analysis allows the determination of the genus and analysis of the phage receptor-binding protein, which interacts with the bacterial receptor and thus influences the host range. Such bacterial receptors include components on the *Salmonella* surface, like outer membrane proteins, and carbohydrates, such as lipopolysaccharide (LPS). While literature on receptors of *Salmonella* Dublin phages is limited, phages infecting other *Salmonella* serotypes use membrane proteins such as BtuB, TolC, OmpC, and various parts of LPS as receptors (Gao et al., 2022; Martinez-Soto et al., 2024). Examining the co-evolutionary dynamics of *Salmonella Enteritidis* and lytic phages showed a strong linear correlation between resistance and phage adsorption, highlighting receptor mutations as the primary reason for phage resistance development (Chen et al., 2024; Barron-Montenegro et al., 2022). However, phages can evolve to overcome bacterial resistance by gene mutations related to the tail proteins responsible for phage binding to receptors, indicating counter-resistance development in the phage (Barron-Montenegro et al., 2022).

## Impact on the microbiota and safety

An advantage of the high specificity and narrow host range of phages is that, unlike broad-spectrum antimicrobials, they selectively target the pathogen while leaving the surrounding commensal microbiota largely unaffected (Palma and Qi, 2024). A healthy rumen microbiota is central to the welfare and productivity of cattle. The microbial population in the rumen is responsible for the fermentative digestion that provides cattle with energy and protein. The interplay of the microbiota comprising diverse microbes, including bacteria, fungi, protozoa, and phages, creates a complex ecosystem (Tardiolo et al., 2025; Herdt, 2020a; Herdt, 2020b), and disruption may have significant consequences for the animal, underlining the advantage of phages which selectively target a specific pathogen while sparing beneficial microbiota, unlike broad-spectrum antimicrobials (Bardina et al., 2012; Alomari et al., 2021). Notably, a study concluded that a *Salmonella* phage cocktail did not affect the microbiota of the gastrointestinal tract of pigs (Thanki et al., 2022). Furthermore, a comprehensive diversity of phages has been identified in the ruminant gastrointestinal tract, with most being lytic with a narrow host range (Wu et al., 2024). This natural occurrence of phages in the ruminant gastrointestinal tract suggests that phage therapy is ecologically compatible with the host and supports its safety, as therapeutic applications would build on microbial interactions that already exist in the environment. Studies have identified a diverse phage community in the ruminant gastrointestinal tract. While some reports suggest that lytic phages with a narrow host range dominate (Wu et al., 2024), others found that lysogenic phages may outnumber the lytic phages (Klieve et al., 1996; Berg Miller et al., 2012). This discrepancy highlights the need for more systematic characterization of the rumen virome. Still, in either case, the consistent presence of phages indicates that they are a natural and ecologically integrated component of the rumen microbiota and may be considered safe.

## Developing efficient and safe phage cocktails

Phages are commonly isolated from environments where their bacterial hosts thrive, such as wastewater, sewage, and retail meat. While no phage cocktail specific to *Salmonella* Dublin has been developed, phages lysing this serovar were previously used for *Salmonella* subtyping (Smith, 1951; Lilleengen, 1950). Furthermore, candidates targeting different *Salmonella enterica* serotypes have been described (Martinez-Soto et al., 2024; Cortes-Ortega et al., 2024; Tao et al., 2021; Zheng et al., 2024; Wang et al., 2022; Zhu et al., 2022; Park et al., 2022; Rivera et al., 2018). To ensure safety and efficacy, only strictly lytic phages are selected, and genome analysis is used to exclude phages carrying undesirable traits, including genes encoding toxins, antibiotic resistance, or proteins implicated in horizontal gene transfer or allergic reactions (Gill and Hyman, 2010). Because individual phages typically have a narrow host range and bacteria can develop resistance, therapeutic applications rely on combining phages that recognize different receptors. For example, a phage cocktail consisting of five taxonomically diverse phages, targeting four different receptors: the O-antigen on LPS, BtuB, OmpC, and the core carbohydrates of LPS, effectively prevented resistance development in *Salmonella Enteritidis* (Martinez-Soto et al., 2024). Similarly, another study proved that a phage cocktail composed of phages utilizing different receptors delays the emergence of resistance compared to single phages (Gao et al., 2022). Importantly, developing resistance to multi-receptor cocktails often comes with fitness costs, such as an increased susceptibility to certain antibiotics (Gao et al., 2022). No cocktails exist for *S.* Dublin, but strategies from other *Salmonella* serotypes provide a framework for design, now strengthened by modern molecular tools, which can optimize phage cocktails. Here, tail fiber engineering by gene swapping allows phages to infect new hosts (Ando et al., 2015), CRISPR-Cas-assisted editing enables precise genome modifications (Martel and Moineau, 2014), and directed evolution can broaden host range (Lin et al., 2025).

## Phage stability

For a phage cocktail to be efficient, the individual phages must survive the physiological conditions during applications, such as adverse temperatures and pH. Temperature is relevant for applications, storage, and production of phage cocktails. For example, a two-phage cocktail survived pelleting used in feed production, showing that phages may be administered to feed for prophylactic use, like vitamins are today (Thanki et al., 2022). Phage titers were stable in feed at 4 °C, whereas they reduced over time in feed at barn temperatures (10 and 25 °C) and −20 °C (Thanki et al., 2022). For therapeutic application in cattle, phages should be stable at the animal body temperature of 38 to 40.5 °C (Terra and Reynolds, 2020). Among the *Salmonella* Dublin phages characterized in literature, all except MSP1, for which no data are available on stability at 4 °C, are stable between 4 °C and 37 °C, indicating good prospects for use in cattle (Table 1). Two phages are stable at 50 °C, while the remaining exhibit a decline in viability at this temperature. Depending on the application, the pH levels at different anatomical sites are also relevant for phage viability. The highest stability among the phages was at pH 5–8 (Zheng et al., 2024; Wang et al., 2022; Zhu et al., 2022; Park et al., 2022) (Table 1), which would enable it to survive in the bovine venous blood and rumen, represented by pH levels between 7.31–7.53 and 5.56–5.56, respectively (Herdt, 2020a; Herdt, 2020b). While the breadth of stability varies between phages, all the phages tested at a pH below 3 showed low viability, thus only causing challenges if the phages reach the abomasum, having a pH of 2 (Herdt, 2020a; Herdt, 2020b).

**Table 1:** Characteristics of phages infecting and killing *Salmonella* Dublin.

Phage	Isolation site	Taxonomy	Phage stability				Phage stability											
			Temperature (°C)				pH											
			4	25	37	50	<3	3	4	5	6	7	8	9	10	11	11<	References
SP154	Raw sewage from a poultry farm Zhangjiagang City, China	Family: *Schittoviridae* Genus: *Ithacavirus*	+	+	+	−	−	−	−	+	+	+	+	+	+	−	−	Zheng et al. (2024)
SP76	Domestic sewage in a sewage treatment plant Nanjing, China	Family*: Demerecviridae* Subfamily: *Markadamsvirinae*	+	+	+	−	−	+	+	+	+	+	+	+	+	−	−	Wang et al. (2022)
PHB12	Sewage from pig farms in Hubei province, China	Family*: Straboviridae* Subfamily: *Tevenvirinae* Genus: *RB69virus*	+	+	+	+	ND	+	+	+	+	+	+	+	−	−	−	Zhu et al. (2022)
MSP1	Mix of environmental samples from poultry meat, feces, organs, soil and sewage, South Korea	Family: *Demerecviridae* Genus: *Epseptimavirus*	ND	+	+	+	−	−	+	+	+	+	+	+	+	−	−	Park et al. (2022)

+ A survival rate above 60% or no discernible reduction in phage titer after 60 min for phages SP154, SP76, PHB12 and 2 h for phage MSP1. Phage titer and survival rate were determined by counting surviving phages by the double-layer agar plate method after incubation at the temperatures and pH-levels depicted above.

− A survival rate below 60% in phage titer after 60 min for phages SP154, SP76, PHB12 and 2 h for phage MSP1. Phage titer and survival rate were determined by counting surviving phages by the double-layer agar plate method after incubation at the temperatures and pH-levels depicted above.

ND, Not determined.

## Clinical considerations

In clinical settings, it’s crucial to consider the intended outcome and method of administration when introducing new technology or medication. Different administration routes offer unique benefits and drawbacks for phage applications and therapy. Among others, successful application routes described in literature include intravenous (Loponte et al., 2021), topical (Kim et al., 2024), intraperitoneal (Gao et al., 2022; Sun et al., 2025), and oral (Thanki et al., 2022; Tao et al., 2021). The choice of administration route will depend on the infection site, practicalities such as storage, and the end goal of treatment. Successful phage therapy was demonstrated by enhanced survival rates of mice infected with *Salmonella Typhimurium* after treatment with phage ZK22 (Sun et al., 2025). Also, prophylactic administration of feed pellets containing a two-phage cocktail caused a significant reduction in *Salmonella* colonization in the gut and fecal shedding of piglets (Thanki et al., 2022). However, phage cocktails are not always successful, as one study describes how the intradermal inoculation of a phage cocktail against *Salmonella* Montevideo was followed by successful delivery to lymph nodes. Despite high efficiency *in vitro,* the expected reduction in *Salmonella* levels was not proven, possibly due to internalization in bacterial cells like macrophages, leading to a loss of phage viability (Wottlin et al., 2022).

*Salmonella* Dublin’s intracellular survival complicates therapy as the phages must cross the host cell membrane; however, some phages can do so. For example, a lytic *Salmonella*-phage isolated from wastewater samples in China was shown to lyse intracellular *Salmonella* in macrophages (Tao et al., 2021). Furthermore, T4 phages can be taken up by mammalian cells and most remain active after internalization (Bichet et al., 2021). However, the internalization rate varies depending on size and phage genus (Bichet et al., 2021; Fajardo-Lubian and Venturini, 2023). Novel strategies to optimize the efficiency of phages against *Salmonella* and other intracellular bacteria are being developed, including but not limited to nanocapping (Meng et al., 2022), engineering of phages (Fajardo-Lubian and Venturini, 2023; Zhao et al., 2023), synergetic combination with antibiotics (Fajardo-Lubian and Venturini, 2023), and liposome formulations (Fajardo-Lubian and Venturini, 2023; Yan et al., 2021; Colom et al., 2015). The latter may help overcome the stability and internationalization in mammalian tissues.

## Discussion

As the threat of antibiotic resistance increases, the need for alternative solutions like phages grows. Applying phage cocktails to treat *Salmonella* Dublin infections could reduce mortality and morbidity rates, helping farmers to mitigate significant economic losses (Nielsen et al., 2013). When used prophylactically, phage applications may limit the appearance of new clinical cases and ensure that infected animals shed fewer bacteria. This will also reduce the prevalence of carriers by eliminating the low concentration of *Salmonella* Dublin, which represents a risk factor for developing carrier animals (Nielsen et al., 2004). Used for decontaminating carcasses, meat, or milk, phages could minimize the risk of *Salmonella* Dublin spreading from cattle to humans as a foodborne pathogen. Combining these with the directions described in the legislation, for example, the Danish eradication program (Ministeriet for Fødevarer LoF, 2022), would significantly contribute to eradication efforts in cattle herds, and a general decrease in contamination in food will lead to fewer human cases.

Yet, all these benefits rely on the development of efficient phage cocktails. While the literature specifically addressing *S.* Dublin is limited, many studies have focused on other serotypes, such as *Salmonella Enteritidis* (Chen et al., 2024; Barron-Montenegro et al., 2022), and different animal hosts (Thanki et al., 2022; Sun et al., 2025). Interestingly, recently described broad-spectrum phages like SP154 and MSP1 could lyse multiple serotypes, including *S.* Dublin (Zheng et al., 2024; Park et al., 2022), highlighting that some phages may cross serotype boundaries. Whether such phages or those characterized in other systems can be applied effectively against *S.* Dublin remains uncertain, as differences in phage receptors, epidemiology, and disease dynamics may influence their activity. Nonetheless, findings from related serotypes provide valuable insights into phage-host interaction and coevolution that can guide the rational design of phage cocktails targeting *Salmonella* Dublin.

The formulation and application of an efficient phage cocktail should ideally be guided by thorough clinical considerations about the physiological conditions in the animal, specific properties of the bacteria, and practical concerns such as production and storage. Different key aspects of stability are relevant, depending on the use of the phage cocktail. If the cocktail is applied to meat, carcasses, or other products to limit zoonotic transmission in the food chain, the phages should be stable at temperatures in abattoirs and refrigeration, as found for all the reviewed phages tested at these temperatures (Zheng et al., 2024; Wang et al., 2022; Zhu et al., 2022; Park et al., 2022), If the cocktail will be used in animals as a therapeutic or prophylactic agent, the chosen phages should be stable at the animal’s normal body temperature. All the investigated phages were stable at 37 °C (Zheng et al., 2024; Wang et al., 2022; Zhu et al., 2022; Park et al., 2022), suggesting their potential use in mammals. Considering that cattle have a slightly higher body temperature (Terra and Reynolds, 2020), it would be helpful to measure stability at the exact temperature for use in cattle. Mainly because some phages showed a steep decline in viability at the following measuring point (50 °C) (Zheng et al., 2024; Wang et al., 2022), and even more so, if the cocktail is intended for use in animals with clinical symptoms of salmonellosis, such as high fever. Another critical aspect of environmental stability is the survival rate of phages at different pH levels. The highest survival rates were between pH 5–8 (Zheng et al., 2024; Wang et al., 2022; Zhu et al., 2022; Park et al., 2022), which aligns well with the conditions found in the rumen and cattle blood (Herdt, 2020a; Herdt, 2020b; Terra and Reynolds, 2020), which looks promising for clinical use. Yet, the low viability of the phages at a pH below 3 may complicate oral administration due to the low pH in the abomasum, the final stomach compartment in cattle (Herdt, 2020a; Herdt, 2020b). Currently, methods to alleviate this challenge are being developed and tested. For instance, it has been shown that bacteriophages encapsulated in liposomes, spherical vesicles consisting of one or more phospholipid bilayers, were less prone to acid damage and had a longer intestinal retention time than non-encapsulated phages (Yan et al., 2021; Colom et al., 2015). When tested against *Salmonella* infection in a broiler model, the encapsulation increased the protection of the phages (Colom et al., 2015).

For *Salmonella* Dublin, the intracellular nature is of some concern for treatment therapy. However, phages were shown to internalize with variation in the rate by size and phage type (Bichet et al., 2021). This property would be beneficial to test before including a bacteriophage in a cocktail against *Salmonella* Dublin. A low internalization rate may require considering application and optimization strategies to be developed and implemented as part of the treatment (Meng et al., 2022; Zhao et al., 2023; Yan et al., 2021; Colom et al., 2015). Research on rumen microbiota suggests that using a bacteriophage cocktail in pigs does not harm their digestive microbial communities (Alomari et al., 2021; Thanki et al., 2022). Yet, it is essential to recognize the differences between monogastric animals like pigs and ruminants such as cattle. Still, the narrow host range of phages, combined with the presence of lytic phages in the gastrointestinal tract of ruminants, indicates minimal risk to rumen health. This distinction sets phage therapy apart from traditional antibiotics, making it a promising alternative that may offer unique benefits.

Interestingly, the high clonality of *Salmonella* Dublin within a geographic area (Kudirkiene et al., 2020; Fenske et al., 2019; Leekitcharoenphon et al., 2023) poses an excellent opportunity for phage treatment, as phages may be able to infect an extensive host range if isolated for a specific region. Other considerations are essential for developing an efficient phage cocktail. For instance, the development of bacterial resistance (Barron-Montenegro et al., 2022; Chaturongakul and Ounjai, 2014) to phages may pose a problem for the efficacy of phage therapy, and, while it has shown that this resistance can come at a cost for the bacteria (Gao et al., 2022; Chen et al., 2024), it is still uncertain how this development would affect grand scale use and whether it is possible to anticipate and counteract this. More knowledge about phages, such as their specific receptors, could deepen the understanding of resistance and allow us to create phage cocktails that safeguard against resistance. Other aspects of phage therapy would be beneficial to explore further, including but not limited to the pharmacokinetics of phage cocktails, specific receptors of individual phages, the most efficient dosages and administration methods, and the development of solid manufacturing processes. As the basis of knowledge about phages grows, so does the potential of their use.

## Conclusion

Despite ongoing eradication efforts, *Salmonella* Dublin is a zoonotic bacterium that causes severe human infections and significant economic losses in the agricultural sector. Phage cocktails may support eradication programs by targeting and killing *Salmonella* Dublin. Cocktails have already been successfully developed and tested against other serotypes of *Salmonella*. By screening for receptors and designing cocktails that focus on complementary interactions, effective phage cocktails can be created with minimal risk of developing resistance. This resistance may be linked to increased susceptibility to other phages or antibiotics.

Certain clinical conditions associated with *Salmonella* Dublin infections could affect the effectiveness of phage cocktails. However, these challenges can be addressed through careful selection of phages, appropriate storage methods, application techniques, encapsulation, and newly developed optimization strategies for phage therapy. To fully harness the potential of bacteriophages in combating *Salmonella* Dublin, the production and application of these cocktails should rely on a thorough understanding of each phage’s characteristics and their interactions with hosts and the environment. Research in this area could promote the design of efficient and safe phage cocktails to combat *Salmonella* Dublin, ultimately benefiting humans and animals.
